# Novel marker-based cone-beam CT registration for enhanced accuracy in dental implant planning

**DOI:** 10.1093/dmfr/twag041

**Published:** 2026-06-05

**Authors:** Maryam Buholayka, Rama Zouabi, Santiago F Cobos, Aditya Tadinada

**Affiliations:** Department of Biomedical Dental Sciences, College of Dentistry, Imam Abdulrahman Bin Faisal University, Dammam, 34212, Saudi Arabia; Department of Oral Health and Diagnostic Sciences, Division of Oral and Maxillofacial Radiology, University of Connecticut, Farmington, CT 06030, United States; Oral Radiology Division, Department of Dentistry, Schulich School of Medicine and Dentistry, Western University, London, ON N6A 3K7, Canada; Department of Oral Health and Diagnostic Sciences, Division of Oral and Maxillofacial Radiology, University of Connecticut, Farmington, CT 06030, United States

**Keywords:** dental implant planning, limited-view CBCT, marker-based registration, digital dentistry, image-guided interventions

## Abstract

**Objectives:**

Accurate image registration is essential for successful digital dental implant planning. Surface-based registration, commonly used in clinical software, is sensitive to cone-beam CT (CBCT) artifacts and relies on manual landmark selection, potentially reducing accuracy. Marker-based registration offers improved precision but is underused due to its technical complexity and time demands. The purpose of this ex-vivo study was to evaluate the accuracy of a novel marker-based registration method using limited-view CBCT reconstructions derived from 2 orthogonal projection images and to compare it with a conventional surface-based registration method.

**Methods:**

An ex-vivo model consisting of a maxillary arch with removable 3D-printed teeth and a radiographic guide containing radiographic markers was used to simulate 5 metal artifact conditions. Full-view CBCT scans were acquired for each condition. For the conventional method, 3 examiners independently performed dual-scan registrations using user-defined surface landmarks on full CBCT reconstructions. For the proposed method, examiners selected orthogonal projection images where markers were clearly visible and limited-view reconstructions were generated to identify marker centroids. Registration error was measured as the point-to-point distance between corresponding surface reference points, standardized to a common frame of reference. Inter-rater reliability was also assessed.

**Results:**

Both methods maintained consistent performance across all artifact conditions. The proposed method demonstrated greater accuracy, with lower point-to-point registration errors and less spatial variation. Inter-rater reliability was excellent for the proposed method and good for the conventional approach.

**Conclusion:**

The proposed marker-based registration method using orthogonal projection images and limited-view CBCT reconstructions showed higher accuracy than conventional surface-based registration under controlled ex-vivo conditions. Although the workflow currently requires additional examiner input, it may improve precision in dual-scan protocols for implant planning. Future clinical studies and software developments are required to support clinical implementation.

Clinical implications Implementing the proposed marker-based registration approach could improve implant placement accuracy by reducing registration errors. The proposed method has the potential to reduce operator dependence in marker-based registration workflows for dual-scan protocols; however, these findings were demonstrated under ex-vivo conditions and require clinical validation.

## Introduction

Cone-beam CT (CBCT) is central to modern dental implant planning, especially when combined with 3D intraoral scanning and CAD-CAM workflows.^1^ CBCT-guided implant surgery requires accurate registration between a digital wax-up and the CBCT-derived 3D alveolar ridge.^2^ This depends on precise identification of fixed reference points between datasets.^3^ These can be anatomical structures or radiographic markers visible across modalities. In CBCT and micro-CT imaging, intensity-weighted centroid localization of radiographic markers is widely accepted as the most reliable method due to its sub‑voxel precision (0.1-0.2 voxels), robustness to noise and artifacts, and optimal performance in minimizing alignment errors.^4^ Accurate marker localization enables reliable digital implant planning.^3^ Two registration techniques are commonly used: (1) surface-based and (2) marker-based registration.

Surface-based registration (SBR) relies on the dental arch geometry of an intraoral scan, or similar, and corresponding points on a digitally generated arch geometry derived from segmented CBCT data. Both 3D datasets are mesh files usually saved in STL file format. Surface-based registration is achieved by minimizing the spatial discrepancies between corresponding geometric features, typically using iterative algorithms.^5^ Surface-based registration does not require radiographic markers and is, generally speaking, faster and less expensive than marker-based registration (MBR).^5^ However, SBR may underperform in certain clinical scenarios, such as edentulous arches or cases with a sparse number of remaining teeth. Surface-based registration is also susceptible to inaccuracies, particularly in the presence of CBCT artifacts from metallic restorations, as highlighted by Biun et al.^3^ These artifacts can slow down the clinical workflow and potentially compromise alignment precision.

MBR, on the other hand, involves the use of radiopaque markers (fiducials) linked to the patient’s anatomy, typically using a radiographic guide. In these protocols, an intraoral surface scan might not be required, instead, 2 CBCT scans are acquired (dual-scan registration): one where the patient is wearing the radiographic guide (guide-on scan) and a second scan of the radiographic guide alone (guide-only scan).^6^^,^^7^ The distinct geometry, and high contrast of the markers makes them easily distinguishable in both scans. However, labeling corresponding markers can be time-consuming and challenging, as they often require operators to manually identify and select fiducial points within CBCT volumes. Once identified, their precise 3D coordinates can be recorded and used to align the datasets with sub-voxel accuracy.^4^^,^^8^ The radiographic guide incorporates clinically relevant information (eg a wax-up) that aids with dental implant planning. But, its fabrication adds further time and cost to the procedure.^8^

Any registration method can produce errors that misposition implants, affecting outcomes.^3^ As stated by Flügge et al,^2^ guided implant surgery, although a great advancement, suffers from deviations between planned and actual implant positions, underlining the need for more accuracy. In SBR, metal artifacts can severely impact the shape and quality of dental surfaces derived from CBCT data.^3^ Current research is focused on reducing metal artifact intensity mainly using iterative reconstruction algorithms, or using different AI-powered strategies.^9–11^ MBR focuses on precise 3D localization of radiographic markers in CBCT, but manual labeling—often in axial CT views—is time-consuming and technically challenging.^12^ Limited-view CBCT reconstruction, using only projections that improve the target’s quality, can reduce noise and artifacts by excluding compromised views.^13^ Similarly, Roentgen stereophotogrammetric analysis (RSA) uses 2 calibrated radiographs to triangulate marker positions.^14^ The potential of fiducial-marker identification utilizing 2 orthogonal, CBCT projection images for MBR in dental implant planning has yet to be investigated.

The objective of this study is to describe and evaluate the use of limited-view CBCT reconstructions derived from 2 orthogonal X-ray projection images to aid in the identification of radiographic marker centroids in the presence of metal artifacts. To investigate this approach, 3 examiners, all final-year residents in oral and maxillofacial radiology, performed dual-scan registrations using a widely established surface-based registration technique^15^ and the proposed 2-view MBR method. Five ex-vivo simulations with varying levels of metal-induced imaging artifacts were designed to evaluate the robustness of each method. The null hypothesis is that there will be no significant difference in dual-scan registration error between methods.

## Methods

Two main objects were fabricated for this ex-vivo study: (1) a plastic maxillary arch with tooth sockets and removable 3D-printed teeth (simulation model) and (2) a radiographic guide containing three 2.3 mm diameter radiopaque markers (CT-Spot; Beekley Medical). The simulation model included 2 sets of 11 attachable 3D-printed teeth. One set included amalgam restorations–occlusal restorations in posterior teeth and buccal restorations in anterior teeth. The radiographic guide featured 4 anchoring roots that enabled secure and reproducible attachment to the simulation model (Figure 1).

**Figure 1:**
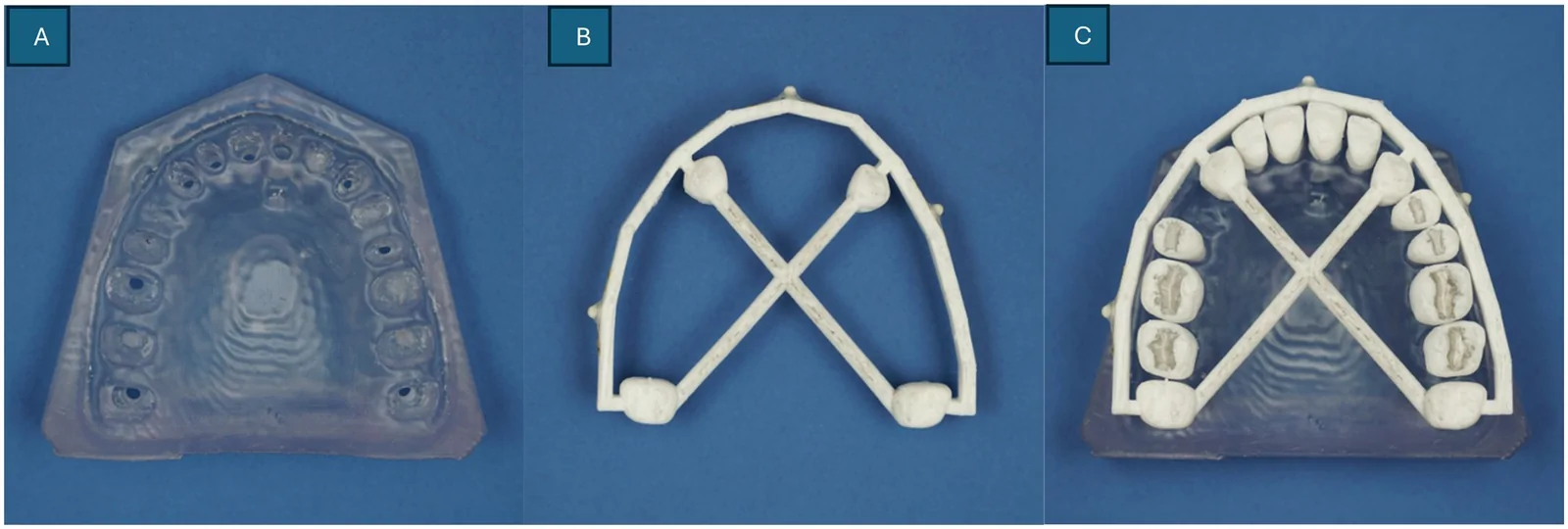
Experimental-setup objects. (A) Maxillary arch simulation model. (B) Radiographic guide with markers. (C) Simulation model with amalgam restored teeth, coupled to radiographic guide.

Assembly of restored and unrestored teeth with the simulation model allowed for configuration of 5 scenarios with varying metal artifact intensity levels (no-artifact, minimal, mild, moderate, and severe) (Figure 2). The artifact intensity levels were defined based on the number of amalgam restorations present, rather than on a quantitative radiographic metric: 0 for the no-artifact condition, 1 restoration for minimal artifacts, 2 restorations for mild artifacts, 4 restorations for moderate artifacts, and 11 restorations for severe artifacts. For each artifact intensity level, the simulation model attached to the radiographic guide was imaged using a CBCT scanner (3D Accuitomo 170; J. Morita Mfg. Corp.) with a 360° degree protocol, 60 kVp, and 7 mA; the total exposure time for each scan was 17.5 s. X-ray tube parameters were modified from the standard clinical protocol to account for the lack of soft-tissues and other anatomical structures. Scans were acquired with a 140 × 100 mm field-of-view. CBCT reconstructions were generated using the unit’s proprietary reconstruction software (i-Dixel 2.3.5.6; J. Morita Mfg. Corp.).

**Figure 2:**
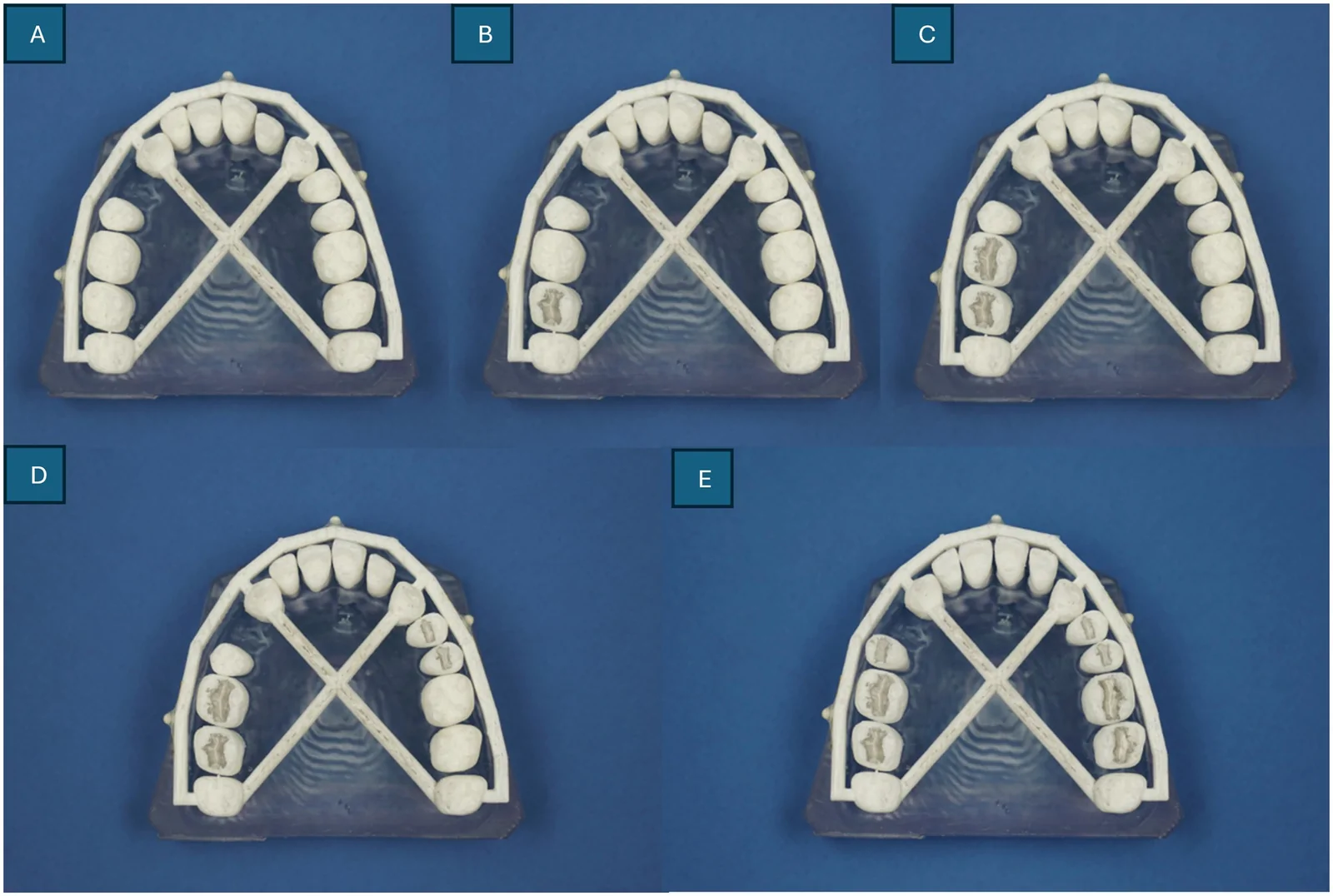
Metal artifact intensity-level setups. (A) No-artifact. (B) Minimal. (C) Mild. (D) Moderate. (E) Severe.

Two additional scans of only the radiographic guide were acquired with the guide in 2 distinct positions (Figure 3A). These scans are referred to as guide-only Position A (G-A) and Position B (G-B). Scan G-A was used to generate a high-resolution and high-accuracy 3D surface model of the guide (G-A_STL_), and to obtain the most accurate, at least under the constraints of the technology, 3D coordinates of the markers (G-A^1^_xyz_, G-A^2^_xyz_, and G-A^3^_xyz_) (Figure 3B). Similarly, Scan G-B was used to obtain the coordinates of corresponding markers G-B^1^_xyz_, G-B^2^_xyz_, and G-B^3^_xyz_. For both scans, these coordinates were calculated using 3D Slicer 5.4 (slicer.org) following a well-established weighted centroid algorithm.^16^^,^^17^ This method uses a threshold value to obtain 3D segmentations of the markers and then the center of mass of the segmented voxels was mathematically calculated. Since this methodology is highly replicable, only operator M.B. calculated the centroids of markers on the G-A and G-B CBCT data. Using the Fiducial Registration Module of 3D Slicer, a registration matrix (GA-to-GB) was calculated to move the G-A_STL_ 3D surface to the G-B scan frame of reference. This process was done to account for any digitization, or computational errors inherent in applying the registration matrix to the G-A_STL_ reference. Additionally, 13 vertices of the G-A_STL_ surface were labeled across the arch and used to measure registration error following an approach similar to Biun et al^3^ (Figure 3C). Therefore, the G-B coordinate system, and G-A_STL_ surface selected points were used to standardize and measure the output of each tested registration method.

**Figure 3:**
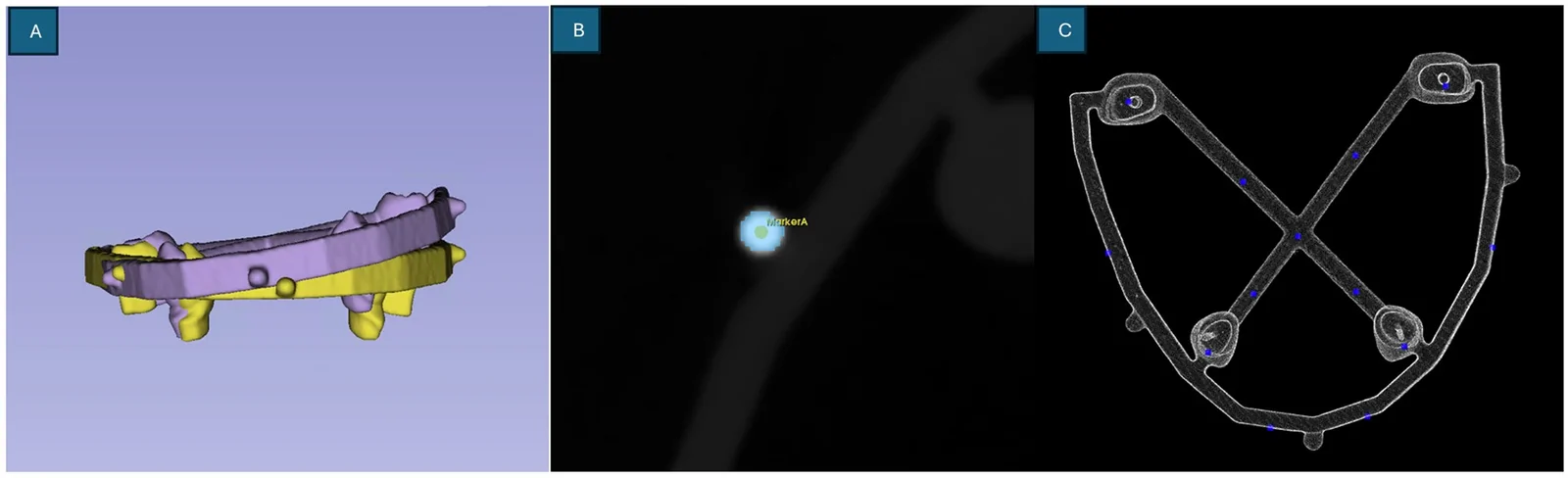
Guide-only scans data. (A) 3D renderings of guide-only scans in Positions A (tilted) and Position B (horizontal). (B) Axial view of segmented radiographic marker (blue) and mathematically calculated centroid (yellow). (C) Wire-frame 3D rendering of the guide-only Position A (G-A_STL_) surface file and the 13 reference points labeled (blue-dots).

In clinical practice, dual-scan registrations aim to superimpose data from a guide-only scan into a guide-on scan. Commonly used registration methods apply user-defined surface landmarks based on threshold segmented 3D surfaces derived from CBCT data.^15^ Clinical software for this purpose usually presents a split screen where clinicians can see 3D renderings of both scans and are prompted to select at least 3 matching surface points. In this ex-vivo study, a similar approach was followed, where the guide-only data were derived from scan G-A, and the guide-on scans derived from the full-view CBCT reconstructions of the simulation model with 5 metal-artifact intensity levels. After a calibration session, the 3 examiners (M.B., R.Z., S.F.C.) selected the 3 radiopaque markers embedded in the radiographic guide as corresponding points for registration using the 3D Rendering and Markups modules in 3D Slicer. Figure 4 shows representative 3D renderings of the no-artifact and advanced artifact scans. For the G-A reference scan examiners selected matching points only once. Five transformation matrices for registration were calculated using the Landmark Registration module of 3D Slicer for each examiner for each G-A and artifact-case pair.

**Figure 4:**
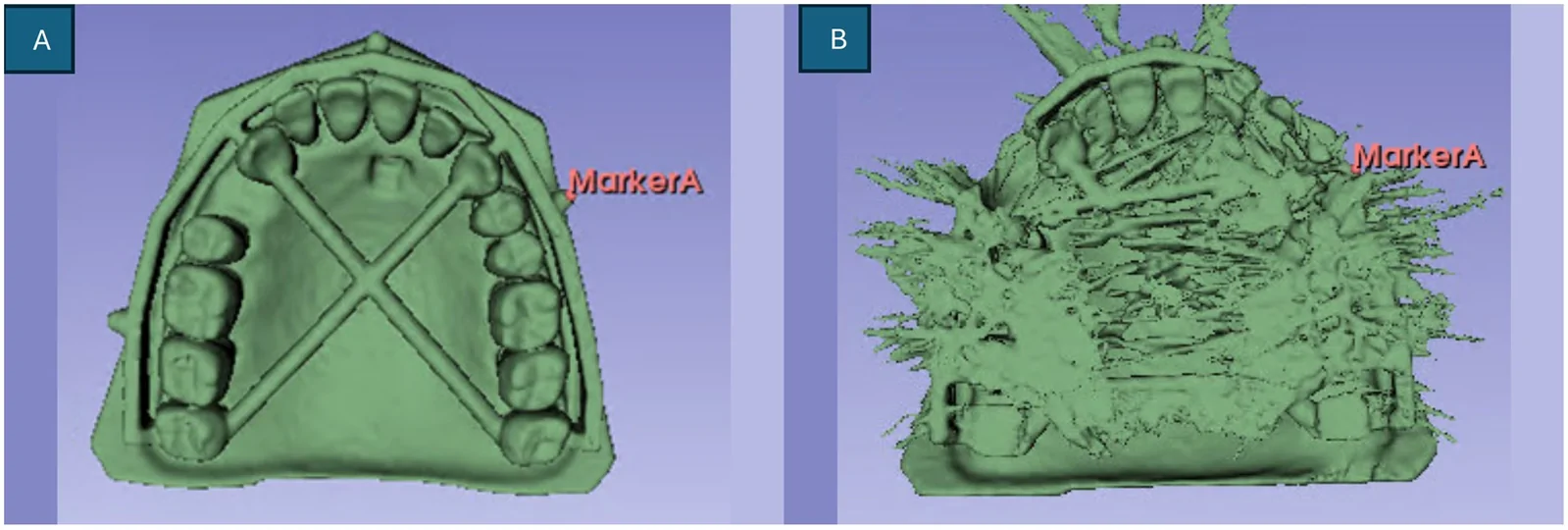
Conventional full-view CBCT simulation cases data. 3D renderings of threshold surfaces for the no-artifact (A) and severe (B) artifact simulation cases. Both images depict the position of 1 user-defined marker for registration.

For the proposed method, examiners were instructed to identify pairs of approximately orthogonal X-ray projection images for each radiographic marker in each scan–selecting views where markers were clearly visible and minimally overlapped by other structures (Figure 5A, B, D, and E). The 579 raw projection images, per scan, were imported into ImageJ v1.54f (National Institutes of Health) as an image stack, which could be navigated using the integrated slider. A custom macro script enabled quick navigation between orthogonal views, with an angular offset of approximately 90 degrees, ∼100 projection images for the Accuitomo 170 unit. If a selected orthogonal view presented significant structural overlap, examiners were instructed to choose an alternative projection from a nearby angle. Each examiner documented their selected projection image pairs. On each projection image, the examiner used the Elliptical Selection tool in ImageJ to define a region of interest (ROI) with a diameter approximately twice that of the visible outline of the marker. An in-house script then applied a 10-pixel gradient blend to merge the selected marker data with a pre-captured bright-field image, generating synthetic projection images. The marker attenuation values were also enhanced by 30% to improve visibility. These synthetic projection images were then merged with a corresponding bright-field stack and imported into i-Dixel for limited-view CBCT reconstruction using the default back-projection algorithm. The resulting volumes depicted the back-projected intersections of each marker (Figure 5C and F). These limited-view reconstructions were loaded into 3D Slicer, where the marker intersections were manually demarcated using the fiducial (point annotation) tool. Figure 6 illustrates the projection image selection, marker extraction, limited-view reconstruction, and centroid localization steps used in the proposed method. Centroid coordinates were then used to compute 5 transformation matrices per examiner, for each guide-only (G-A) and artifact-containing scan pair, using the Landmark Registration module in 3D Slicer.

**Figure 5:**
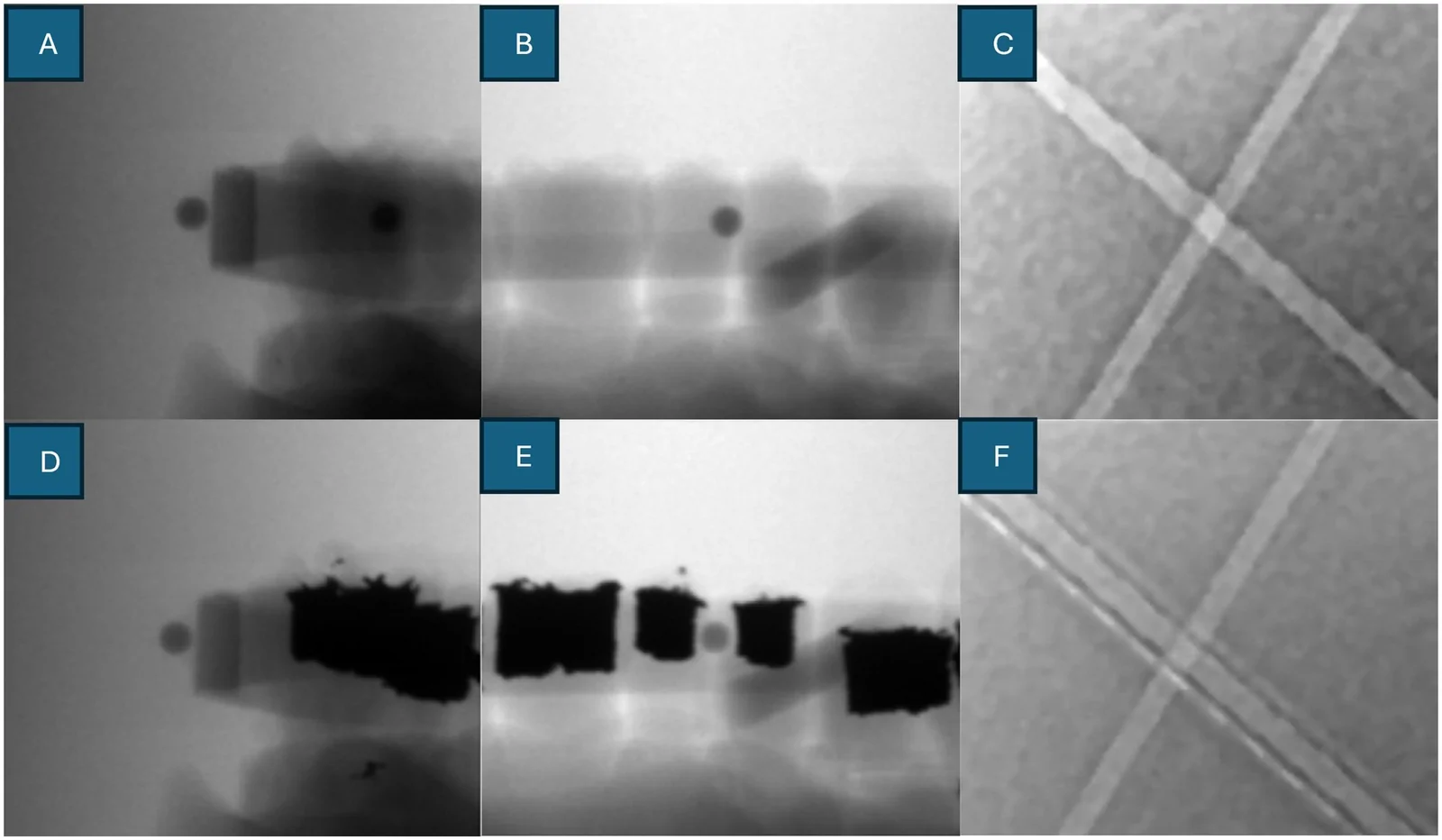
Proposed method simulation cases data. Top row: no-artifact case scenario. Bottom row: severe artifact case scenario. (A, B, D, and E) Magnified projection images of radiographic markers. C and F, magnified corrected-axial view of limited-view CBCT reconstructions.

**Figure 6:**
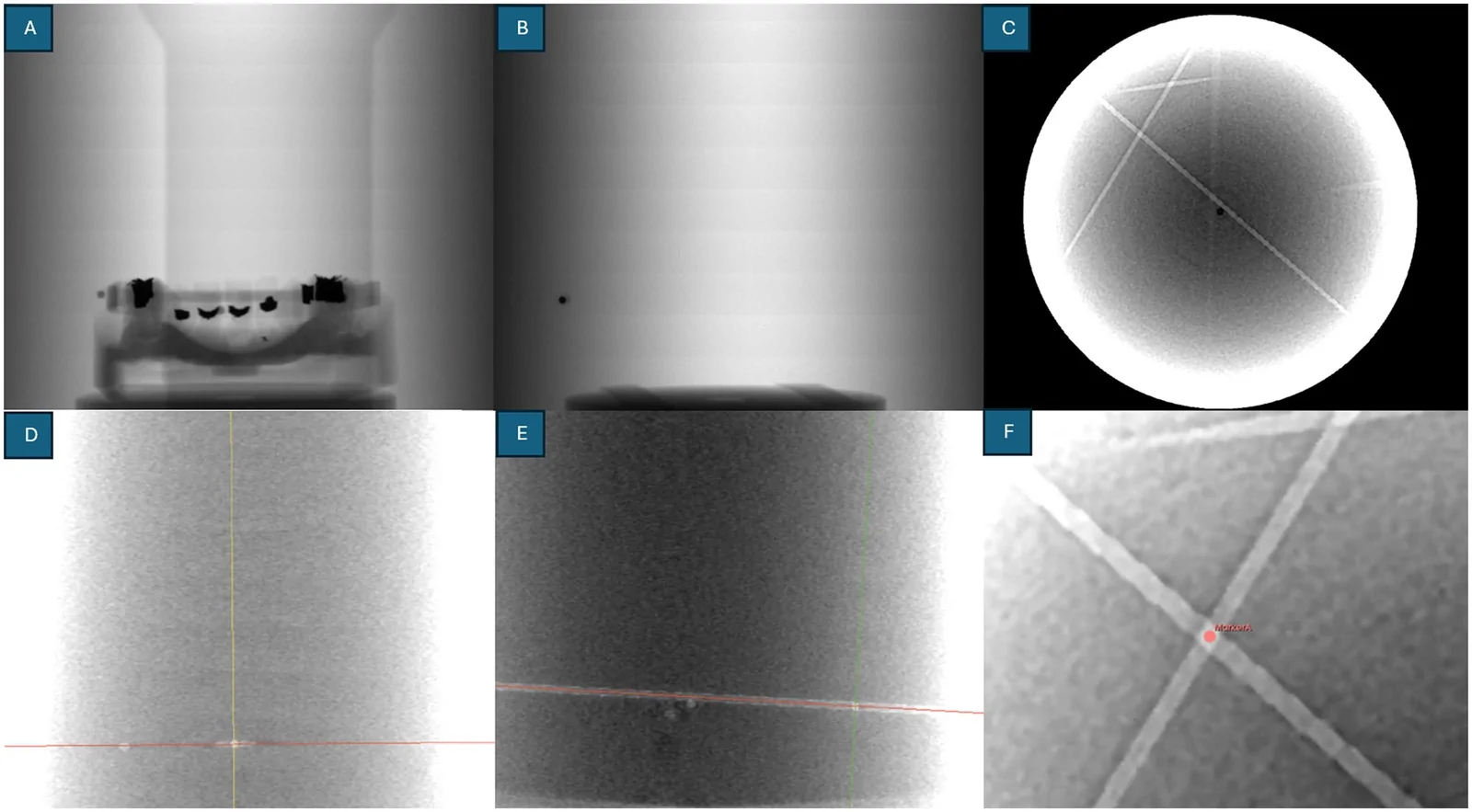
Illustration of the marker demarcation process used in the proposed method. (A and B) Selection of example projection image and extraction of the radiographic marker region using the elliptical ROI tool in ImageJ, followed by merging with a pre-captured bright-field image to generate a synthetic projection image. (C) Limited-view CBCT reconstruction in 3DSlicer demonstrating the back-projected marker region. (D and E) Orthogonal views in 3DSlicer used to identify the marker intersection corresponding to the radiographic marker location. (F) Marker centroid localization performed in 3DSlicer using the fiducial (point annotation) tool.

Transformation matrices are used to register a “moving” object into the coordinate system of a “fixed” object by aligning corresponding sets of 3 or more 3D coordinates. In dual-scan protocols, it is typical to transform the guide-only scan into the coordinate frame of the guide-on scan. However, because rigid transformations are invertible, registration error remains equivalent in magnitude regardless of which scan is designated as the fixed or moving object.^18^ Leveraging this principle, and to standardize outputs across the 2 evaluated registration methods, the following “4-step” process was implemented. (1) The reference surface file (G-A_STL_) was first transformed into the coordinate system of the guide-on scan (G-B), using matrix GA-to-GB. (2) A duplicate of the G-A_STL_ file was then transformed into the coordinate system of the simulated scenario using the matrix GA-to-conventional (or GA-to-proposed, depending on the method under evaluation). (3) The transformed file was returned to its original coordinate system (G-A) using a transformation matrix derived exclusively from the evaluated registration method (conventional-to-GA or proposed-to-GA). (4) Finally, the duplicated surface was moved into the G-B coordinate system using GA-to-GB. Table 1 illustrates the various registration matrices used in this study. A schematic overview comparing the conventional surface-based registration (SBR) workflow and the proposed orthogonal marker-based registration (MBR) workflow used in this study is shown in Figure 7.

**Figure 7:**
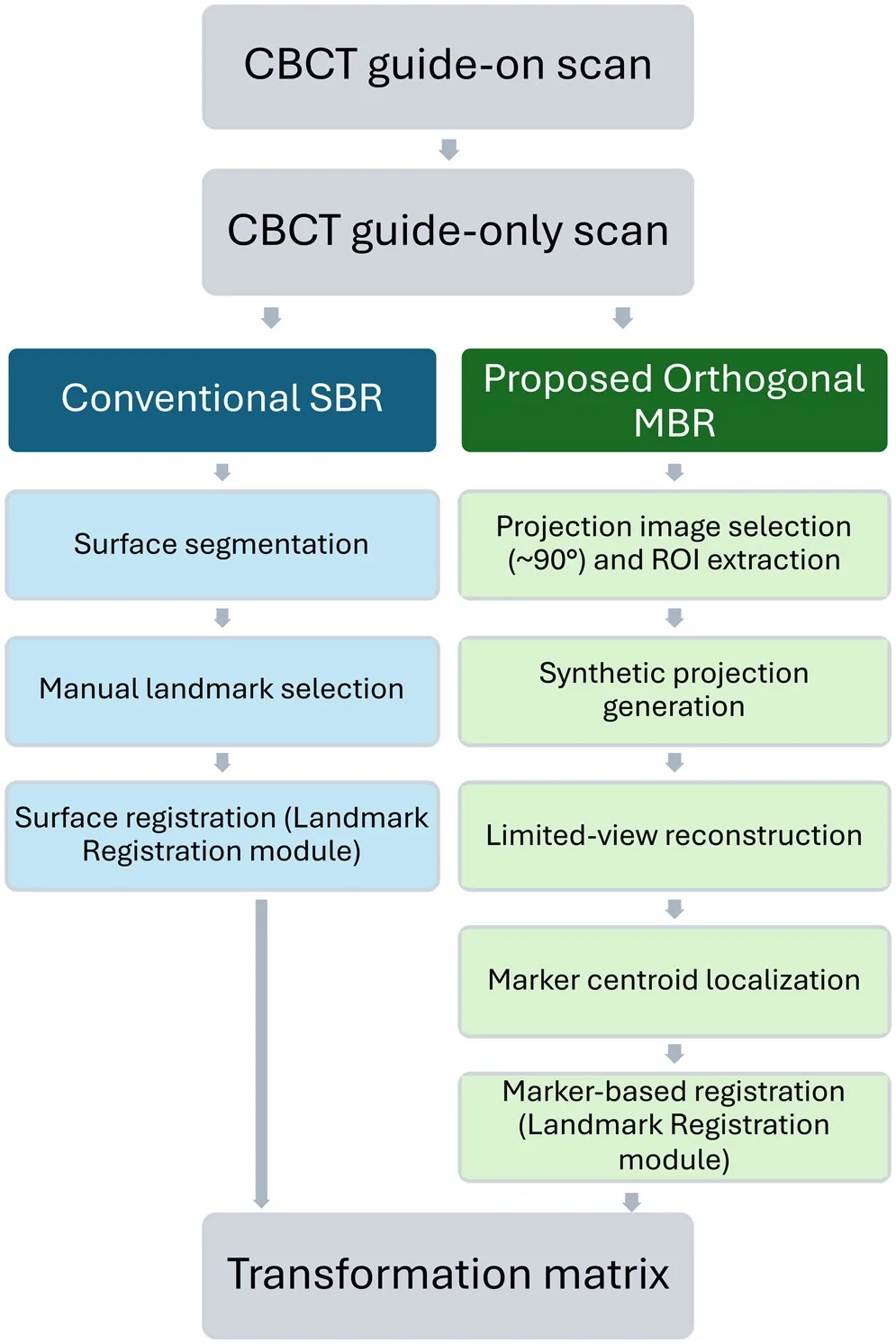
Conceptual workflow comparing the conventional surface-based registration (SBR) pipeline and the proposed orthogonal marker-based registration (MBR) pipeline used in this study. Both methods begin with guide-on and guide-only CBCT acquisitions but differ in how corresponding registration points are identified prior to computing the transformation.

**Table 1:** List of data, methods for marker identification, and corresponding registration matrices to evaluate the conventional and proposed registration methods under the no-artifact simulation scenario.^a^

Moving data	Fixed data	Moving markers- method	Fixed markers-method	Registration matrix name
**G-A/FV**	G-B/FV	WC	WC	GA-to-GB
**G-A/FV**	NA/FV	WC	CR	GA-to-conventional^NA^
**G-A/FV**	NA/2V	WC	PR	GA-to-proposed^NA^
**NA/FV**	G-A/FV	CR	CR	conventional^NA^-to-GA
**NA/2V**	G-A/FV	PR	PR	proposed^NA^-to-GA

a Abbreviations:2V = two-view reconstruction; CR = conventional-registration method; FV = full-view reconstruction; G-A = guide-only Position A; G-B = guide-only Position B; NA = no-artifact simulation scenario; PR = proposed registration method; WC = weighted-centroid method.

The registration error was quantified as the absolute distance between corresponding reference points in the 2 G-A_STL_ files (original vs duplicate), computed using the Cloud-to-Cloud measuring tool in CloudCompare 2.12.4 (cloudcompare.org). Because registration errors can vary spatially, the mean error for each marker was calculated and visualized using a color scale in the 3D viewer of CloudCompare to qualitatively assess spatially dependent error trends.

Statistical software (IBM SPSS Statistics v23.0; IBM Corp) was used for all statistical tests. Normality of the data distribution was assessed using the Kolmogorov–Smirnov and Shapiro–Wilk tests. The data were found to be normally distributed. The unit of analysis was the point-to-point registration error measured across all reference points and artifact conditions. The inter-rater reliability for both the conventional and the proposed method was assessed using the intraclass correlation coefficient (ICC) score. These scores were interpreted using Koo and Li guidelines.^19^ The effects of artifact intensity levels within each registration method were evaluated using a one-way analysis of variance (ANOVA). Finally, a paired Student *t*-test was used to evaluate differences in registration outcome between the conventional and the proposed registration methods.

## Results

All examiners successfully completed the required steps for both registration methods (conventional and proposed) across all 5 simulated scenarios with varying levels of metal artifact severity. For the proposed method, examiners were required to select 2 orthogonal slices. Angular equivalents ranged from 90 to 101 degrees, with 1 examiner consistently selecting a 90 degree angle across all cases.

There were no statistically significant differences in registration point-to-point error across artifact levels within either method. The average distance error for the conventional method was 0.18 mm (SD = 0.077 mm). The proposed method showed an average distance error of 0.18 µm (SD = 0.08 µm). The difference in average distance error between the 2 methods was statistically significant (*P* < .0001, paired Student *t*-test), as summarized in Table 2. The error values, in millimeters, for each artifact level and for both methods are illustrated in Figure 8.

**Figure 8:**
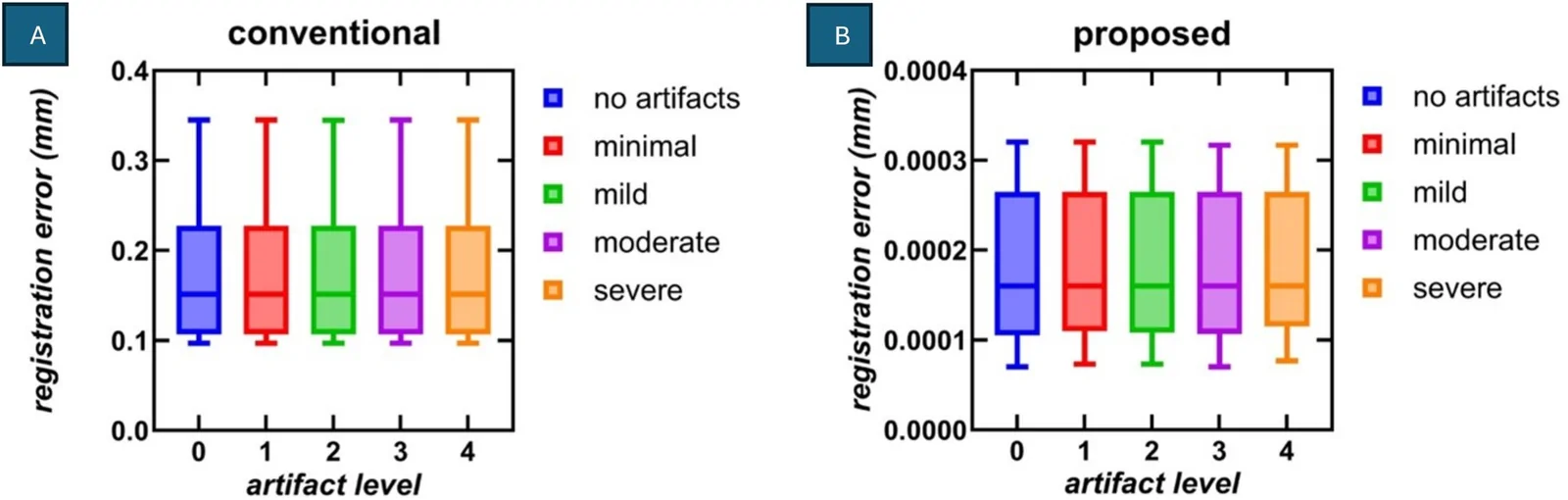
Registration error results. Average registration errors across metal artifact simulated scenarios. (A) conventional method. (B) proposed method.

**Table 2:** Overall comparison of registration error between conventional and proposed methods.^a^^,^^b^

Method	Mean (mm)	±SD
**Conventional**	0.175482	0.0765924
**Proposed**	0.000180	0.0000810

a Student *t*-test Paired: *P < .0001; Highly significant.

b Mean difference: 0.175 mm.

For the conventional method, mean error values ranged from approximately 0.175-0.176 mm across all artifact scenarios. For the proposed method, mean error values ranged from approximately 0.000178-0.000182 mm across the same artifact conditions. One-way ANOVA demonstrated no statistically significant effect of artifact intensity on registration error for either the conventional method (*F* = 0.000, *P* = 1.000) or the proposed method (*F* = 0.003, *P* = 1.000) (Table 3).

**Table 3:** Mean registration error across artifact intensity levels for the conventional and proposed registration methods.^a^

Artifact level	Conventional method mean ± SD (mm)	Proposed method mean ± SD (mm)
**Absent**	0.175 ± 0.079	0.00018 ± 0.00008
**Minimal**	0.176 ± 0.079	0.00018 ± 0.00008
**Mild**	0.175 ± 0.079	0.00018 ± 0.00008
**Moderate**	0.175 ± 0.079	0.00018 ± 0.00008
**Severe**	0.176 ± 0.079	0.00018 ± 0.00008

Values represent ± SD (mm).

a Artifact intensity did not significantly affect registration error for either method (one-way ANOVA: conventional method *F* = 0.000, *P* = 1.000, proposed method *F* = 0.003, *P* = 1.000).

The mean point-to-point error for all 13 markers combining all examiners and all simulated scenarios for each registration method are shown in Table 4. A color-coded axial view of these mean errors, in millimeters, for both registration methods is illustrated in Figure 9. Reference points are labeled with the same nomenclature used in Table 4. For the conventional registration method, reference Point 2 had the largest error value (0.35 mm) and Point 9 the lowest error value (0.10 mm). For the proposed registration method, reference Point 10 had the largest error value (0.0003 mm) and Point 1 the lowest error value (0.0001 mm). Descriptive comparison across the 13 reference points demonstrated spatial variability in registration error for the conventional method, whereas the proposed method showed consistently minimal variation across points.

**Figure 9:**
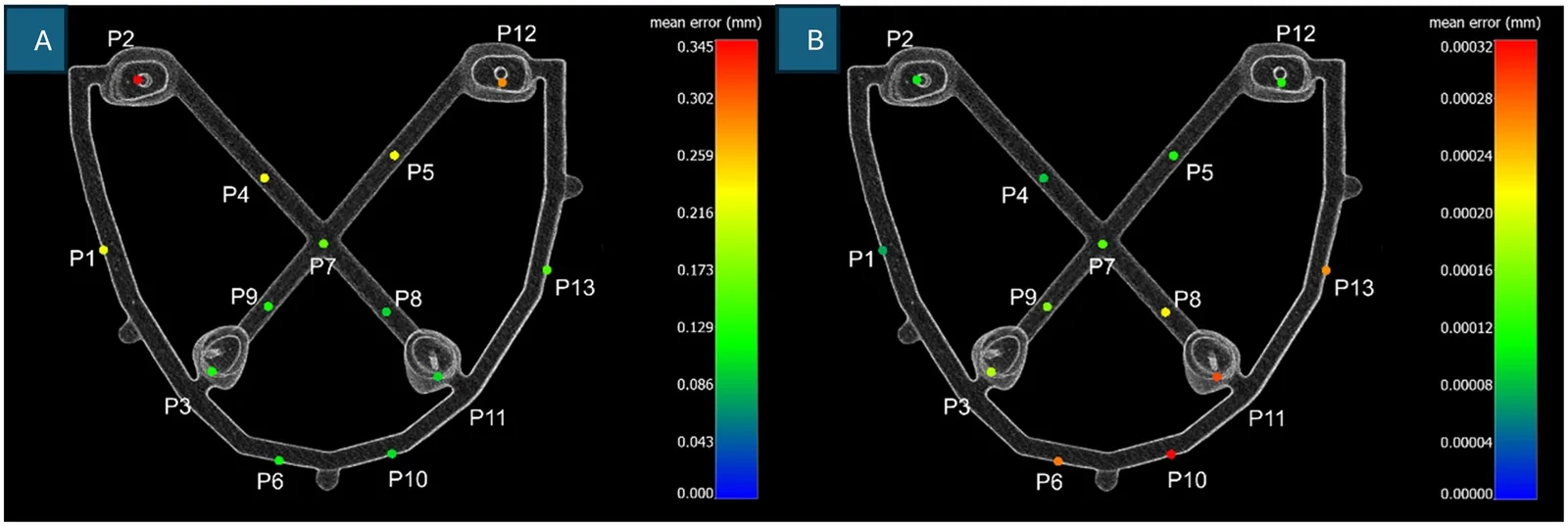
Spatial distribution of mean registration errors. Axial view of the STL surface file and 13 reference points. (A) Color-mapped mean registration errors for the conventional method. (B) Color-mapped mean registration errors for the proposed method.

**Table 4:** Mean point-to-point registration errors for the 13 reference points for both the conventional and proposed methods.^a^

	Conventional (mm)	Proposed (mm)
**Point 1**	0.2243	**0.0001**
**Point 2**	**0.3451**	0.0001
**Point 3**	0.1248	0.0002
**Point 4**	0.2292	0.0001
**Point 5**	0.1264	0.0002
**Point 6**	0.1124	0.0003
**Point 7**	0.1598	0.0001
**Point 8**	0.2258	0.0001
**Point 9**	**0.0971**	0.0002
**Point 10**	0.1012	**0.0003**
**Point 11**	0.1008	0.0003
**Point 12**	0.2828	0.0001
**Point 13**	0.1515	0.0003
**Mean**	0.1755	0.0002

a Values represent mean error (mm) across all examiners and simulated scenarios. Bold values indicate the lowest and highest errors within each method.

The ICCs for both the conventional and proposed methods were 0.88 (95% CI, 0.82-0.93) and 0.997 (95% CI, 0.996-0.998), respectively, indicating good reliability among examiners for the conventional method, and excellent reliability among examiners for the proposed method.

## Discussion

Although CBCT projection images are rarely used beyond reconstruction in dentistry, this study shows their potential in dual-scan registration. A method using selected projections to localize radiographic markers was evaluated, comparing it to a commonly used surface-based registration method. Sub-voxel, intensity-weighted centroid registrations from full-view CBCT volumes were employed as reference. Instead of repeated single-operator measures, multiple examiners were tested to capture inter-operator variability, which can affect clinical accuracy. Both methods showed strong inter-rater reliability, with the proposed method demonstrating slightly better consistency, suggesting its potential as an operator-independent registration tool. In conventional surface-based registration, operators must manually identify corresponding surface landmarks across 2 complex 3D datasets, which may introduce variability depending on experience and interpretation of anatomical features. In contrast, the proposed method relies on identifying radiopaque markers with high contrast in projection images, which are easier to visually isolate and therefore less sensitive to subjective interpretation. In addition, the centroid of each marker is calculated automatically using an intensity-weighted algorithm, which further reduces manual variability in marker localization.

The simulated dual-scan scenarios included 5 varying levels of metal artifacts as these have been reported as one of the main sources of registration error when working with CBCT data in digital implant planning.^1^^,^^3^^,^^6^ The results demonstrated that both registration methods performed consistently across all scenarios. This might be attributed to limitations in the ex-vivo simulations which did not include additional sources of CBCT artifacts (eg scatter radiation from soft tissues). Scatter radiation can significantly increase the severity and distribution of CBCT metal artifacts.^20^ Additionally, examiners performed conventional registrations in order of complexity from the no-artifact to the severe artifact scenarios, which could have introduced performance bias when selecting surface features in the 3D models. Another alternative is that the radiographic guide design, with markers placed a few millimeters away from the tooth surface, minimized the shape altering effects of metal artifacts. In a clinical setting, radiographic guides and markers are designed to rest directly on the tooth surface.^21–23^ Regardless of these considerations, the proposed method is likely to be less susceptible to metal artifacts if the selection of orthogonal images for limited-view CBCT reconstructions ensures minimal structural overlap.

Another critical factor in achieving accurate dual-scan registration is minimizing spatially dependent discrepancies by ensuring a well-distributed placement of radiographic markers across the anatomy of interest.^24–26^ For example, a second molar may be registered with less accuracy than anterior teeth if markers are not positioned sufficiently posteriorly. The results from the conventional surface-based registration method suggest susceptibility to such errors, as the largest deviations in registration error were observed in regions of the guide farthest from the radiographic markers (Points 2 and 12 in Figure 9A). These discrepancies may be clinically relevant, with registration errors approaching ∼0.3 mm. In contrast, the proposed method exhibited a linear gradient in registration deviation from left to right and posterior to anterior, corresponding to the tilted positioning of the guide in the guide-only Position A scan (Figure 3A). These minor deviations are likely attributable to digitization artifacts, a known limitation of weighted centroid algorithms.^17^ However, given the minimal registration error observed (∼0.0003 mm), these discrepancies are unlikely to have clinical implications.

A practical limitation of marker-based registration methods that use underlying CBCT data for weighted centroid calculations, which provide sub-voxel accuracy, is the time-consuming nature of manually selecting matching markers in CBCT multi-planar reconstructions. The proposed method simplifies this task by using projection images for marker identification and selection. However, the workflow still involves multiple processing steps across different software platforms, which may limit reproducibility outside the authors’ environment due to variability in user interaction and software configurations. The very low error values observed (0.0001-0.0003 mm) should be interpreted in the context of computational precision and controlled experimental conditions. These values are below the intrinsic spatial resolution of CBCT systems and likely reflect the use of intensity-weighted centroid calculations on high-contrast markers, combined with the absence of patient-related variability such as motion and soft tissue scatter. Although in this study the process was tedious, future software development could automate key steps, including projection selection and marker identification, reducing operator input and improving reproducibility for clinical implementation.

The proposed method may also be susceptible to motion artifacts. Centroid calculation accuracy could be compromised if patient movement occurred between the selected projection images. This issue might be mitigated by reviewing the projection image stack for signs of motion and discarding images acquired after the motion event.

Another limitation of this study is the absence of an independent physical reference standard, such as micro-CT. The present design focused on comparing 2 registration workflows under controlled conditions using a consistent computational reference framework. Although centroid localization derived from full-view CBCT reconstruction provides a stable internal reference, future investigations should validate the proposed approach against independent ground truth measurements and under conditions that more closely resemble clinical imaging environments, including the presence of soft tissues and additional sources of scatter radiation.

In summary, the proposed method demonstrated higher accuracy than the conventional surface-based technique under the tested conditions. While the traditional method matched prior reports (0.1-0.3 mm)^3^, the proposed approach had minimal registration error. This suggests orthogonal projection images may improve dual-scan registration. Further research will assess its feasibility and benefits for routine dental implant planning workflows.

## Conclusion

Based on the findings of this ex-vivo study, the following conclusions were drawn:

1. Limited-view CBCT reconstructions can provide sufficient detail to identify the 3D centroids of radiographic markers, with accuracy comparable to that of full-view CBCT reconstructions and weighted centroid algorithms.
2. Using projection images to identify radiographic markers may reduce registration errors in dual-scan protocols for image-based digital dental implant planning; however, this finding requires validation in clinical settings.
3. Registration based on landmark matching in 3D surface models demonstrated lower accuracy than the newly investigated method using orthogonal projection images and was more susceptible to spatially dependent registration errors under the conditions of this study.

## Data Availability

The data underlying this article will be shared on reasonable request to the corresponding author.
